# A randomized controlled trial of self-management for people with epilepsy and a history of negative health events (SMART) targeting rural and underserved people with epilepsy: a methodologic report

**DOI:** 10.1186/s13063-021-05762-z

**Published:** 2021-11-20

**Authors:** Gena R. Ghearing, Farren Briggs, Kristin Cassidy, Michael Privitera, Carol Blixen, Martha Sajatovic

**Affiliations:** 1 Department of Neurology, Carver College of Medicine and University of Iowa Health Center, Iowa City, IA USA; 2 Department of Population and Quantitative Health Sciences, Case Western Reserve University School of Medicine, Cleveland, OH USA; 3 Department of Psychiatry & of Neurology, Case Western Reserve University School of Medicine and University Hospitals Cleveland Medical Center, Cleveland, OH USA; 4 UC Gardner Neuroscience Institute, University of Cincinnati College of Medicine, Cincinnati, OH USA; 5 Department of Psychiatry & Neurological and Behavioral Outcomes Center, Case Western Reserve University School of Medicine, Cleveland, OH USA

**Keywords:** Epilepsy, Seizure, Self-management, Rural, Hospitalizations, Emergency room

## Abstract

**Background:**

Many people living with epilepsy (PLWE) reside in rural communities, and epilepsy self-management may help address some of the gaps in epilepsy care for these communities. A prior randomized control trial of a remotely delivered, Web-based group format 12-week self-management program (SMART) conducted in Northeast Ohio, a primarily urban and suburban community, demonstrated improved outcomes in negative health events such as depression symptoms and quality of life. However, there is a paucity of research addressing the needs of PLWE in rural settings.

**Methods:**

The present study leverages collaboration between investigators from 2 mid-western US states (Ohio and Iowa) to replicate testing of the SMART intervention and prioritize delivery to PLWE in rural and semi-rural communities. In phase 1, investigators will refine the SMART program using input from community stakeholders. A Community Advisory Board will then be convened to help identify barriers to trial implementation and strategies to overcome barriers. In phase 2, the investigators will conduct a 6-month prospective randomized control trial of the SMART program compared to 6-month waitlist controls, with the primary outcome being changes in negative health events defined as seizure, self-harm attempt, emergency department visit, or hospitalization. Additional outcomes of interest include quality of life and physical and mental health functioning. The study will also assess process measures of program adopters and system end-users to inform future outreach, education, and self-management strategies for PLWE.

**Discussion:**

The method of this study employs lived experience of PLWE and those who provide care for PLWE in rural and underserved populations to refine a remotely delivered Web-based self-management program, to improve recruitment and retention, and to deliver the intervention. Pragmatic outcomes important to PLWE, payers, and policymakers will be assessed. This study will provide valuable insights on implementing future successful self-management programs.

**Trial registration:**

ClinicalTrials.gov NCT04705441. Registered on January 12, 2021

## Background

It is estimated that one in 26 Americans will develop epilepsy which remains associated with a high burden of medical complications, decreased quality of life, and premature mortality [1]. A growing body of research supports self-management approaches that can improve outcomes in people living with epilepsy (PLWE). However, because of diverse needs across various sub-populations and known health disparities, it is important to understand how evidence-based epilepsy self-management approaches might impact outcomes across a broad range of PLWE, including those living in rural and semi-rural communities.

While the literature on disparities in epilepsy is growing, comprehensive, comparative data remain limited [2], and most of the literature has not focused on the needs of rural communities. Twenty percent of the US population reside in rural communities, which tend to have a higher rate of poverty, depression, and suicide than urban communities [3, 4]. There has been a widening gap in healthcare outcomes referred to as the rural mortality penalty, resulting in over 134 excess deaths per 100,000 [5]. A survey of Midwestern neurologists identified low income, lack of insurance, transportation difficulties, stigma, and misperceptions about epilepsy as barriers interfering with epilepsy care in rural regions [6]. Residents of rural communities and other underserved communities may experience health disparities and have multiple areas of unmet need in epilepsy care. A recent targeted review on the social determinants of health in epilepsy recommended engaging patients, especially those from disadvantaged backgrounds, in self-management in order to educate and empower patients [7]. The Managing Epilepsy Well (MEW) Network has been a national leader in developing, testing, and disseminating evidence-based epilepsy self-management programs [8]. One of these, the self-management for people with epilepsy and a history of negative health events (SMART) program, developed by researchers at Case Western Reserve University (CWRU), is an online 12-week behavioral group format program targeted to reducing barriers and maximizing facilitators to self-care in high-risk people with epilepsy [9]. SMART has been demonstrated to reduce epilepsy-related complications and improve quality of life [10]. SMART is delivered by peer educators (patients with epilepsy) and nurse educators. Thus, SMART combines the portability and low cost of a Web-based intervention with the personally salient components of behavior modeling that can be obtained by interacting with individuals who have “walked the walk” in living with and coping with epilepsy.

A new SMART 2.0 research study is intended to (1) replicate efficacy findings of the original SMART randomized control trial (RCT) in an alternative setting (rural and semi-rural communities), (2) incorporate best practices and recommended strategies for enhancing study recruitment and retention, and (3) assess program adopter’s perceptions of the intervention to inform future implementation and dissemination. This report describes the methods of the planned replication study using a prospective 2-site, 6-month RCT design to assess SMART vs. 6-month waitlist (WL) control. The study will assess effects on epilepsy complications and other outcomes in a diverse sample with epilepsy including rural people with epilepsy. We hypothesize that at 6-month follow-up, SMART will be associated with reduced negative health events (NHEs) compared to WL. We also expect that SMART will be associated with improved quality of life, functioning, and physical and mental health.

## Methods

**Fig. 1:**
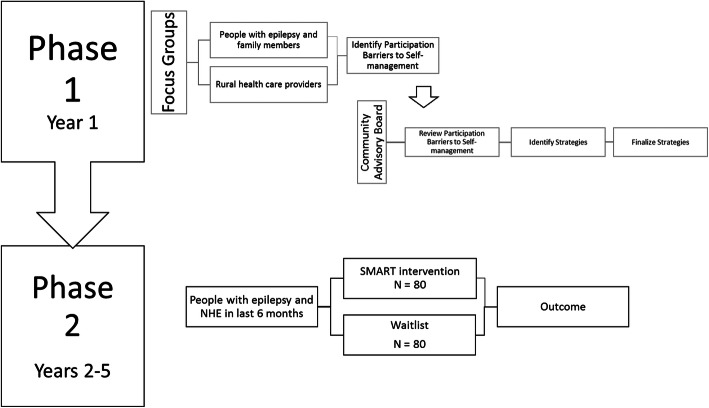
Phase 1 and phase 2 study operationalization. NHE negative health event

This 2-site, 2-phase study (Fig. 1) will investigate the effects of the SMART program on health outcomes in people with epilepsy who have had recent NHEs defined as a seizure, self-harm attempt, emergency department visit, or hospitalization within the last 6 months. The study will be conducted in Ohio and in Iowa, led by a team of researchers from CWRU in Cleveland and from the University of Iowa (UI) in Iowa City in collaboration with stakeholders in epilepsy and rural healthcare.

### Phase 1

Phase 1 is intended to set the stage for a successful RCT that will inform future dissemination and implementation efforts should RCT findings be positive. Qualitative methods will be used to collect information from informed and committed local stakeholders. Two focus groups composed of PLWE and their family members from rural communities in Iowa and two focus groups composed of rural health providers, social services agency administrators/staff, or other professionals working in rural health will be convened to collect information on perceived barriers and facilitators to participation in an epilepsy self-management program, as well as impressions of the SMART self-management curriculum. Information obtained from these local stakeholders will also help the investigators to develop a robust recruitment strategy and a set of practices that will maximize engagement and retention of subjects. Focus groups will be conducted via Zoom technology, last 60–90 min, and use a semi-structured interview guide adapted from previous studies in self-management of neurological conditions conducted by the CWRU investigators [11, 12]. The total number of individuals in the focus groups will be in the range of 32–40 individuals, which is within the recommended sample size range for qualitative studies [13, 14].

### Community Advisory Board (CAB)

Building upon strong existing partnerships between members of the study team and epilepsy/neurological care partners across the states of Iowa and Ohio, the study team will obtain input from relevant community stakeholders to develop an a priori recruitment and retention strategy intended to maximize enrollment of typically hard-to-reach individuals with epilepsy from underserved and rural communities. The CAB will be composed of up to 16 individuals, including individuals with epilepsy, epilepsy specialists, clinicians, and administrators who practice in rural or underserved communities, and representatives from the Epilepsy Association/Epilepsy Foundation.

There will be 3 video-conference calls prior to the RCT. In the first call, CAB members will review the SMART curriculum and identify potential barriers to recruitment and retention. In the second meeting, CAB members will review the list of barriers and suggest solutions and strategies for reaching people with epilepsy who may not be initially help-seeking and discuss potential methods of retaining these individuals in the program once they are recruited. In the third CAB meeting, the list of strategies will be finalized, and the investigators will obtain input on how these strategies might be best operationalized.

### Phase 2

Phase 2 is a prospective 6-month comparative trial of SMART vs. WL in 160 individuals with epilepsy and a recent NHE. Participants will be randomly assigned (1:1 basis) to receive either SMART (*N* = 80) or WL (*N* = 80) using a computer-generated random number generator created in REDCap during the enrollment process. This is a simple randomization scheme together with permuted block randomization to insure similar treatment and waitlist group sizes. This randomization scheme is unavailable to study staff who enroll participants and assign to treatment or waitlist groups. The participants and nurse and peer educators are not blinded, due to the nature of the intervention. However, the data analysts have no interaction with participants or the nurse or peer educators. The investigators will conduct 8–10 separate and sequential SMART groups with up to 10 individuals in each group. SMART will consist of two main components: a 12-week remotely accessed “intensive” group format stage and a 12-week remotely accessed Web/telephone follow-up stage.

### Setting and recruitment

To assess the potential of SMART to promote behavior change in people with epilepsy and NHEs, the investigators will build on their strong track record of working with local federal, state, and county entities that provide service to rural and underserved communities. At the Iowa site, study participants will be drawn from local clinical and community referral sources including the Epilepsy Foundation of Iowa and primary care networks. At the Ohio site, enrollment will emphasize recruitment outside of the Cleveland metropolitan area with assistance from the Epilepsy Association, Epilepsy Alliance of Ohio, and epilepsy care professionals affiliated with the University of Cincinnati. This will include advertisements and brochures. We will also obtain HIPAA Waivers of Authorization from the relevant IRBs at the recruiting sites so that we may identify and contact potential subjects. Since SMART is an adjunct to ongoing neurological and medical care, it is expected that study participants will continue to receive services with their regular medical clinicians and providers at all sites.

Participants will have a self-reported diagnosis of epilepsy, be at least 18 years of age, have experienced an NHE within the last 6 months, and be able to provide written informed consent and participate in study procedures. Study entry criteria are purposely broad in order to best represent “real world” people with epilepsy [15]. Actively suicidal/homicidal individuals and those with dementia will be excluded, as will pregnant women who are likely to need different and more intensive treatments.

The study team will attempt to oversample PLWE who live in rural or semi-rural settings. Classification of rurality will be determined based upon the 2013 Rural-Urban Continuum Codes [16], a classification scheme that distinguishes metropolitan counties by the population size of their metro area and nonmetropolitan counties by degree of urbanization and adjacency to a metro area. The official Office of Management and Budget metro and non-metro categories have been subdivided into 3 metro and 6 non-metro categories. Each county in the USA is assigned 1 of the 9 codes.

In addition to the RCT participants, the investigators will enroll and train up to 8 peer educators using procedures like those conducted in previous work [17]. A peer educator will be an individual with epilepsy who has had at least 3 previous lifetime NHEs. Informed consent will be obtained for all focus group members, peer educators, and RCT participants, by study staff during the enrollment process. Additional consent must be obtained to include the de-identified subject data in the MEW network database. All work will be conducted consistent with Institutional Review Board (IRB) approval at the relevant sites. Adverse events will be reported to the IRB. Regular meetings between the enrolling sites will communicate any changes in protocol. Annual updates will be provided to the CDC, including any adverse events. This is a low-risk study, but if a participant is identified as suicidal or homicidal during the screening process or at any time during the study, the study staff interacting with the individual will use existing clinical infrastructure to insure safety and appropriate clinical follow-up. The primary investigators will monitor the study to ensure data integrity and the safety of the participants at weekly meetings with study staff. The study biostatistician will review the data for discrepancies after about one-third of participants have been enrolled, at the midpoint of enrollment, and on completion of enrollment. The study data coordinator, who will not be involved in the study intervention and has no contact with participants, will review the study records for compliance with IRB requirements and verification of source documents.

Confidentiality of the research data will be protected in several ways. Paper assessment forms and other study records will be stored in locked research offices. Subjects will be identified by a separate study ID number on all study records. The lists that link study ID codes with subject names and all study records will be stored on password-protected, encrypted computers or secure servers. Only aggregate data will be presented or published and will be presented such that individual patients cannot be identified. Study personnel sign a pledge of confidentiality as a requirement for employment. All study personnel will be required to be certified in the protection of human subjects throughout the study.

Retention efforts will also be maximized by using known strategies to increase retention, including providing participants with a copy of their study visit schedule including a list of procedures to be completed at each visit, e-mail and telephone visit reminders, and keeping multiple updated forms of contact. Participants who do not participate in a SMART session or a research assessment will be called the same day to have the appointment rescheduled. If problems with adherence arise, we will explore with the participant the potential obstacles and ways to improve adherence. After the first year of the project, CAB meetings will be held annually to review study progress and suggest additional strategies that might be needed should recruitment or enrollment be less than expected targets.

Upon project completion, the investigators will present findings to the UI Prevention Research Center, our project-specific CAB, and the CDC MEW Network Coordinating and Collaborating Centers and to community health partners. Findings will be presented at relevant scientific venues and in peer-reviewed publications and may help to scale-up epilepsy self-management program opportunities. We will contribute our de-identified data to the MEW Network database so that it can be shared with a larger audience of scientists interested in epilepsy self-management. Individuals outside of the MEW Network have the opportunity to access MEW data, under appropriate data-sharing agreements, by presenting an analysis proposal to the MEW Database Steering Committee. In addition to the above, we will also post study results on www.clinicaltrials.gov and will make de-identified data available to other qualified investigators in the research community. In all cases, data will be shared as soon as it is available and for as long as the format allows.

### Experimental intervention

The SMART curriculum is divided into 2 steps. Step 1 consists of eight group format 45–60-min sessions with up to 10 participants per group, which will be collaboratively delivered by a nurse educator and a peer educator. The intervention will be delivered in a Web-based format emphasizing interactive discussion with a secure videoconferencing system. Participants will be able to log on and interact via audio and/or video. Telephone call-in will be available for those who do not have Internet access. Patients will receive e-mail and phone call reminders of sessions, and make-up sessions will be available. The initial group-session portion of SMART will be completed over 10–12 weeks (Table 1). Following the step 1 group sessions, step 2 consists of 3 brief (no more than 15 min) monthly Web-based or telephone maintenance sessions conducted by the nurse educator. Telephone sessions will address ongoing issues of epilepsy self-management including treatment adherence. Additionally, the nurse will serve as a facilitator of linkage between the individual’s epilepsy care clinicians by providing SMART program status updates to providers.

**Table 1:**
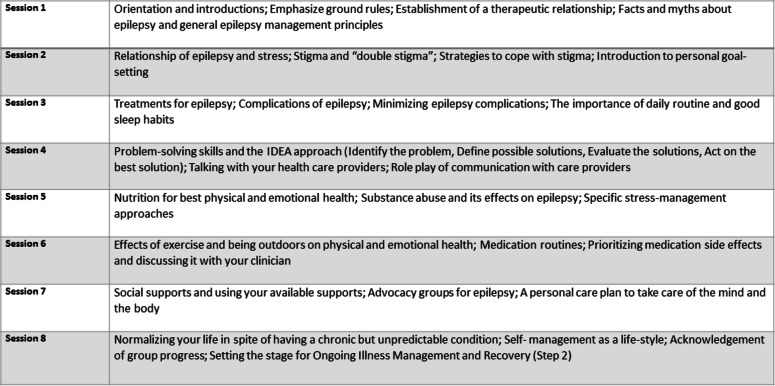
SMART curriculum: topics covered in each session

### Waitlist (WL) control

Individuals randomized to WL will continue in their usual care. After they complete their 13-week and 6-month assessments, which are identical to the SMART group assessments, WL individuals will begin participating in the SMART intervention for the next 12 weeks. Once the WL individuals begin participating in SMART, they will have research assessments at 13 weeks, 6 months, and 12 months after beginning SMART.

### Feasibility and fidelity

Attendance for each SMART session will be recorded. Acceptability will be assessed at the end of each 12-session group series with a brief self-rated survey. Following Fraser [18], fidelity to the SMART intervention will be assessed quantitatively and qualitatively. Non-interventionist research staff will monitor and assess at least 25% of group sessions, with each fidelity dimension being rated on a 1–10 scale.

### Outcomes and analysis

Table 2 outlines study procedures. In addition to demographic and clinical information (age, gender, ethnicity, self-reported cumulative medical illness [19, 20]), health literacy will be assessed [21] to detect persons with limited or marginal health literacy. For both the SMART and the WL groups, research assessments will be done at screening (to establish study eligibility), at baseline, and at 13 weeks, 6 months, 12 months, and 18 months after randomization. Members of the waitlist will also be assessed 13 weeks after starting the SMART intervention. All assessments are suitable for remote administration either by phone or using REDCap (Research Electronic Data Capture), a secure Web application for building and managing online surveys and databases [22]. REDCap will be used for secure data entry and storage.

**Table 2:**
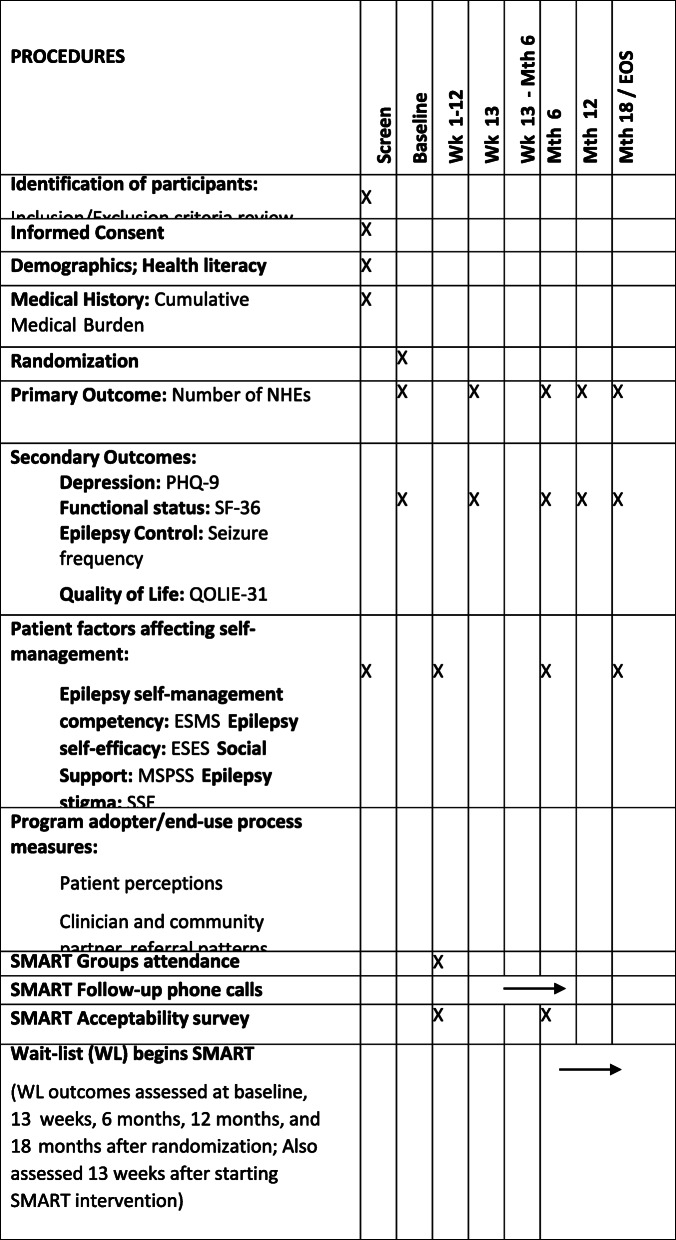
Phase 2/RCT procedure. *Wk* week, *Mth* month, *NHE* negative health events, *EOS* end of the study

#### Primary outcome

The primary outcome will be the proportion of individuals with reduction in their total number of NHEs in the prior 6 months at baseline compared to week 24 of the trial. The prior SMART study had a nonstandard distribution of NHEs and a high proportion of subjects with no NHEs at 6-month follow-up, so a longitudinal binary mixed model (no NHE or at least one NHE) will be used as opposed to a count regression model [10]. The second primary outcome will be the change in NHE counts in the 6 months prior to baseline compared to week 24 of the trial. NHEs are defined as seizures, emergency department visits, hospitalizations, and self-harm attempts. NHE reporting will be via self-report with corroboration by EHR documentation and report of family/support system informants whenever possible. In addition to total NHEs, we will assess NHEs in each category, including seizure frequency and length of stay in days for hospitalization NHEs. These domains (total number of NHEs, seizure frequency, length of hospital stay) will be assessed for differences between the two groups looking at mean and median values. A type I error rate of 0.05 will be considered significant.

#### Secondary/additional outcomes

Additional assessments will include evaluation of depressive symptoms using the PHQ-9, a widely used and validated self-rated depression scale [23]. Functional health status will be assessed using the 36-Item Short Form Survey (SF-36), a multi-purpose, short-form health survey that yields two psychometrically based components: a physical component summary and a mental component summary, which has proven useful for comparing the relative burden of diseases [24]. Epilepsy control will be assessed via self-reported seizure frequency. Quality of life will be assessed with the Quality of Life in Epilepsy (QOLIE-31) self-administered questionnaire which comprises 7 components including seizure worry, overall quality of life, emotional well-being, energy-fatigue, cognitive functioning, medication effect, and social function [25, 26]. These domains (score on PHQ-9, score on SF-36, self-reported seizure frequency, and score on QOLIE-31) will be assessed for differences between the two groups looking at mean and median values at baseline and 13 and 24 weeks of trial and change over time from baseline to 24 weeks of trial. A type I error rate of 0.05 will be considered significant.

Other factors assessed in the trial include epilepsy self-management competency, self-efficacy, social support, and stigma. Self-efficacy will be measured with an Epilepsy Self-Efficacy Scale (ESES) 2000 version. The ESES was developed based on Bandura’s conceptualization of self-efficacy to assess a person’s confidence in his or her ability to use self-management strategies. Previous studies show acceptable construct validity and high test-retest reliability in the ESES [27–29]. Social support will be measured with the Multidimensional Scale of Perceived Social Support (MSPSS), a 12-item scale that measures an individual’s perception of social support provided by family and friends, as well as satisfaction with that support [30]. Stigma for epilepsy will be measured using the 24-item Stigma Scale of Epilepsy (SSE) developed by the Demonstration Project on Epilepsy as part of the WHO/ILAE/IBE Global Campaign against Epilepsy [31, 32]. The SSE is a validated, versatile, and sensitive instrument which has been used mainly in resource-poor settings to study stigma in epilepsy.

### Process measures

Curran and colleagues define process evaluation as a rigorous assessment approach designed to identify potential and actual influences on the conduct and quality of future implementation and dissemination [33]. The process evaluation will target 2 main areas: (1) Identify and describe program adopter’s perception of the SMART program and (2) “Reach” among clinicians and community partners for SMART referral. Patient perceptions will be assessed via a set of open-ended questions regarding their satisfaction with the program, comprising Likert-style questions assessing positive/negative/neutral components of the program and whether they would recommend participation in SMART to a family member or friend with epilepsy. Referral sources (clinicians, community partners, and other referral sources) will be tracked for all study screens as well as successful enrollments.

### Data analysis

Descriptive analyses will compare the individuals assigned to SMART or WL using Student’s *T*-test, Fisher’s exact test, and non-parametric tests when appropriate. The primary outcome is whether there is a difference in the proportion of individuals with reductions in the total number of NHEs in the prior 6 months, at baseline, and at follow-up (6 months after randomization) between SMART and WL, compared using a Fisher’s exact test. We will also test for the difference in NHE counts between baseline and follow-up using a non-parametric Wilcoxon-Mann-Whitney test (the distribution of this measure is expected to be non-normal based on our prior experience). Exploratory longitudinal mixed models with a subject-level random effect will be conducted to investigate a binary variable of no NHEs versus at least one NHE and for a count measure of total NHEs from baseline to 10 and 24 weeks. Explanatory variables will include study site, age, gender, race/ethnicity, number of seizure medications, rural/urban continuum status, and attendance or deviations from the study protocol. Analysis will be performed for the intention-to-treat population (all participants enrolled to receive the SMART trial), for the safety population (all participants who received any portion of the SMART intervention), and for the per-protocol population (all participants who completed the SMART intervention). There will be a delay between randomization and baseline assessments for individuals enrolled for both the SMART trial and the waitlist, which may vary based on the recruitment time required for cohorts and the number of cohorts that can be accommodated at one time. Every effort will be made to insure steady recruitment and sufficient numbers of cohorts to accommodate those recruited. In addition, the specific cohorts will be able to be identified for analysis, and if we find marked variability between the cohorts or the delay in starting the intervention, this will be addressed by the data monitoring committee.

The 12-month and 18-month measurements of NHEs will be compared to baseline and 6-month measurements as well. We will also examine change-over-time in the secondary outcomes using parametric and/or non-parametric tests as deemed appropriate for each measure. NHE measurements will reflect the preceding 6-month time period, except for the 13-week time period. The 13-week NHE measurement will reflect the time period following baseline (when individuals participating in SMART will be having their “intensive” group sessions), and it will be used to assess for within-subject differences from baseline to 13-week and 13-week to 6-month time periods. Note that the 13-week to 6-month NHE count will be derived by subtracting the measurement taken at 13 weeks from the one taken at 6 months. This will allow for a greater understanding of the time course for when the expected reductions occur in the SMART group, which we expect to mostly occur after the “intensive” 8 group sessions have ended. A secondary analysis of NHEs will include a comparison of hospital and emergency department visits. A 2-sided type I error rate of 5% will be considered significant.

### Sample size calculations

Psychosocial interventions can reduce seizures and emergency department visits in people with poorly controlled epilepsy [34, 35]. Oosterhuis noted a 50% reduction in seizures with group psychoeducation [36]. In our original SMART efficacy trial, a total number of past 6-month NHEs were reduced by > 50%. The projected sample size is 160, with 80 subjects per arm. While the Web and phone-based access format of SMART optimized study retention and attrition rate in the original RCT was 14.2%, we conservatively assume 20% attrition at 6 months (*N* = 128). A 2-sided, 2-sample, Fisher’s exact test with a type I error of 5% will have 86% power to replicate the difference in the proportion of individuals with NHE reductions. For the second primary outcome which has a Laplace distribution, a 2-sided, 2-sample, Wilcoxon-Mann-Whitney test with a type I error of 5% will have 89% power to replicate the difference in NHE counts in the prior 6 months.

## Discussion

The study procedures outlined above are intended to perform a SMART efficacy replication trial while working to recruit and retain people with epilepsy in rural and semi-rural communities and to evaluate some of the barriers to self-management particular to rural communities. Rural communities are composed of heterogeneous populations, which have their own needs and vulnerabilities. Many migrant workers and indigenous people live in rural regions, and rural communities have larger proportions of people over the age of 65 and of people with disabilities than urban communities [3]. Many people with epilepsy live in geographically isolated rural communities, and it is important to understand how transportation, access to resources and education, and community attitudes impact epilepsy care and self-management efficacy. Nationwide, there is a shortage of neurologists, but this is especially true in rural communities [37]. It is important to understand how we can best empower people with epilepsy who live in rural communities, and it is possible that self-management may help address some of the gaps in care for rural communities.

Innovative and important aspects of the proposed project include a focus on people with epilepsy who live in underserved and rural communities and use of a lived experience and provider stakeholder group composed of individuals from underserved and rural communities to inform the use of best practices to maximize outreach/recruitment and retention. In addition, this study method allows a focus on pragmatic epilepsy outcomes relevant to both PLWE and payers/policymakers including seizure occurrence, emergency department visits, hospitalizations, and self-harm attempts.

## Trial status

We used protocol version 2 from July 2, 2020, and anticipate starting recruitment for the clinical trial in April of 2021 and completing recruitment by the end of 2023.

## Data Availability

The authors of this paper at UI and CWRU will have full access to the final trial dataset.

## Abbreviations

PLWE: People living with epilepsy
SMART: Self-management for people with epilepsy and a history of negative health events
CWRU: Case Western Reserve University
RCT: Randomized control trial
WL: Waitlist
NHE: Negative health event
UI: University of Iowa
CAB: Community Advisory Board
IRB: Institutional Review Board
REDCap: Research Electronic Data Capture
EHR: Electronic health records
PHQ-9: Patient Health Questionnaire 9-question depression scale
QOLIE-31: Quality of Life in Epilepsy 31-item short form
ESES: Epilepsy Self-Efficacy Scale
MSPSS: Multidimensional Scale of Perceived Social Support
SSE: Stigma Scale of Epilepsy
WHO: World Health Organization
ILAE: International League Against Epilepsy
IBE: International Bureau for Epilepsy
